# The Predictive Effects of Burnout, Academic Buoyancy and Enjoyment on Students’ English Academic Achievement: A fsQCA Approach

**DOI:** 10.3390/bs15111471

**Published:** 2025-10-29

**Authors:** Danjie Sheng, Liping Pu, Honggang Liu

**Affiliations:** 1 School of Foreign Languages, Soochow University, Suzhou 215006, China; 20244204032@stu.suda.edu.cn; 2 Soochow College, Soochow University, Suzhou 215006, China

**Keywords:** English learning burnout, academic buoyancy, foreign language enjoyment, fsQCA, configurational study, the control-value theory

## Abstract

This study investigates how English learning burnout (ELB), academic buoyancy (AB), and foreign language enjoyment (FLE) jointly and independently influence the English academic achievement of Chinese senior high school students. Drawing on the Control-Value Theory of Achievement Emotions, data from 640 students were analyzed using both regression and fuzzy-set Qualitative Comparative Analysis (fsQCA). Regression results indicated that intrinsic enjoyment of language learning was the strongest positive predictor of achievement, whereas exhaustion exerted a notable negative effect. The fsQCA results revealed five pathways to high achievement, such as the combination of high enjoyment and buoyancy with low burnout, which predicted success even without strong teacher support. Conversely, low buoyancy and enjoyment coupled with high burnout characterized underachievement. These findings enrich Control-Value Theory by highlighting asymmetry between the causes of success and failure, and they emphasize the importance of fostering both intrinsic enjoyment and resilience in exam-driven educational contexts. Practical strategies are suggested to help educators reduce negative states and promote sustainable learning engagement.

## 1. Introduction

In second language education, the academic outcomes are shaped by not just the cognitive abilities of the learners, but also by their emotional and motivational experiences. The Control-Value Theory (CVT; Pekrun, 2006) provides a thorough theoretical framework to understand the connections among emotions, motivation, and academic performance. As per CVT, achievement emotions come from the learners’ cognitive evaluations of control (like self-efficacy, perceived competence) and value (such as task importance, interest), which then influence learning engagement, strategy use, and final academic outcomes (Pekrun & Perry, 2014). Recent advancements in educational psychology highlight that achievement emotions—be it positive or negative—play a crucial part in molding students’ engagement, persistence, and ultimate performance (Jin & Zhang, 2021; Liu & Song, 2021; Liu et al., 2025c, 2025d). Within this field, English learning burnout (ELB) (for instance, Liu & Zhong, 2022), academic buoyancy (AB) (af Ursin et al., 2020; Liu et al., 2025d), and foreign language enjoyment (FLE) (Dewaele & MacIntyre, 2014; Jin & Zhang, 2021) have garnered specific scholarly attention.

Burnout in English learning is typically reflected in emotional exhaustion, disengagement, and a declining sense of accomplishment, which can undermine sustained effort and achievement (Li et al., 2023). In contrast, academic buoyancy captures students’ everyday capacity to handle academic stressors and bounce back from setbacks, serving as a psychological shield against burnout (Yun et al., 2018). Enjoyment in language learning, a positive emotional state, strengthens motivation and promotes deeper learning engagement (Dewaele & MacIntyre, 2014).

Although prior research has investigated these variables, most studies have relied on variable-centered methods such as regression or structural equation modeling (Dewaele & Dewaele, 2017; Liu et al., 2025c; Putwain & Wood, 2023). Such approaches often overlook the possibility that distinct combinations of these factors may jointly determine achievement. The fuzzy-set Qualitative Comparative Analysis (fsQCA) offers a useful alternative, as it identifies multiple causal pathways that can lead to the same outcome—a particularly relevant perspective in complicated educational settings (Schneider & Wagemann, 2012).

Within the Chinese senior high school system, where students face intense exam pressure, it is essential to understand how burnout, buoyancy, and enjoyment interact to shape learning outcomes. Addressing this gap, the present study applies fsQCA to examine how different configurations of these psychological variables predict English academic achievement, thereby contributing both to theory building and to the design of targeted pedagogical interventions.

## 2. Literature Review

### 2.1. The Control-Value Theory of Achievement Emotions

The Control-Value Theory of Achievement Emotions (CVT; Pekrun, 2006) provides a comprehensive framework for explaining how emotions emerge from learners’ cognitive appraisals of control (e.g., perceived competence, self-efficacy) and value (e.g., task importance, intrinsic interest), and how these emotions, in turn, shape engagement, self-regulation, and academic outcomes (Pekrun & Perry, 2014). Positive activating emotions such as enjoyment and pride typically arise when learners experience both high control and high value, thereby promoting persistence and deep learning (Pekrun et al., 2002). Conversely, low control and low value often lead to deactivating emotions such as hopelessness or burnout that undermine performance.

While CVT has been widely validated across educational contexts, its explanatory power in second language learning warrants critical refinement. In foreign language settings, emotions are shaped not only by cognitive appraisals but also by social–contextual dynamics such as teacher support, peer relations, and classroom climate (Dewaele & MacIntyre, 2016; Shao et al., 2019). These dimensions extend beyond CVT’s original cognitive-evaluative focus, suggesting that achievement emotions in language learning emerge from an interaction between control–value appraisals and social-relational factors. Thus, to understand affective dynamics in L2 learning, CVT must be expanded toward an integrative socio-cognitive–affective perspective (Liu et al., 2025d; Zhang et al., 2024).

A further theoretical enrichment comes from combining CVT with Self-Determination Theory (SDT; Deci & Ryan, 2000; Ryan & Deci, 2017). SDT posits that motivation and emotional well-being stem from the satisfaction of three basic psychological needs: autonomy, competence, and relatedness. The need for competence parallels CVT’s control appraisal, while the need for autonomy corresponds to the value component, as intrinsically valued tasks satisfy self-determined goals. Relatedness, largely absent in CVT, provides an additional explanatory layer by accounting for interpersonal enjoyment and social support—key affective ingredients in language classrooms (Dewaele & Dewaele, 2017).

The current study builds on and extends CVT in three key ways. First, it reconceptualizes achievement emotions as configurational phenomena rather than linear outcomes. By applying fuzzy-set Qualitative Comparative Analysis (fsQCA), the study demonstrates that distinct combinations of emotional and motivational states—such as high enjoyment with moderate buoyancy and low burnout—can jointly lead to high academic achievement. This approach reveals the asymmetric and equifinal nature of affective influences, challenging CVT’s implicit assumption of symmetric relationships (Fiss, 2011; Woodside, 2013). Second, by incorporating SDT’s autonomy and relatedness dimensions, the study refines CVT’s explanatory scope to encompass socially derived enjoyment (teacher and student support) alongside intrinsic enjoyment (learning itself). Third, the findings empirically illustrate that positive and negative emotions operate in non-mirror ways, confirming that the mechanisms leading to success differ qualitatively from those leading to failure—thus extending CVT through the principle of causal asymmetry (Ragin, 2008; Pekrun & Perry, 2014).

Taken together, this theoretical synthesis positions the present research within an expanded affective-motivational framework that bridges CVT and SDT, aligning emotional control-value processes with need satisfaction dynamics. This integration provides a richer basis for interpreting how English learning burnout, academic buoyancy, and foreign language enjoyment interact configurationally to influence students’ academic achievement.

### 2.2. English Learning Burnout

The concept of English Learning Burnout (ELB) is grounded in the broader construct of academic burnout, which was originally adapted from workplace burnout studies (Maslach & Jackson, 1982). Academic burnout refers to a state of chronic stress in educational settings, characterized by emotional exhaustion, depersonalization or cynicism toward learning, and a reduced sense of personal accomplishment (Schaufeli et al., 2002). Within the context of second or foreign language learning, ELB specifically reflects students’ feelings of fatigue, disinterest, and inefficacy in relation to English study.

English learning burnout emerges when sustained pressures, such as heavy workloads, high-stakes examinations, or low perceived competence, erode students’ intrinsic motivation and enjoyment of learning. As Salmela-Aro and Upadyaya (2014) note, burnout is not a sudden phenomenon but the result of prolonged imbalance between demands and resources. In English learning contexts, this imbalance may manifest in students’ constant struggle with vocabulary acquisition, grammar mastery, or communicative performance, coupled with insufficient coping strategies or support systems.

The structure of ELB is typically conceptualized as a multidimensional construct (Liu & Zhong, 2022). Building on Maslach and Jackson’s (1982) three-factor framework, researchers in educational psychology have identified three core dimensions of burnout: emotional exhaustion, cynicism (or depersonalization), and reduced academic efficacy (Schaufeli et al., 2002). However, Liu and Zhong (2022) recruited 1213 Chinese senior high school students to examine a construct of ELB more suitable for Chinese EFL learners; they reported a two-dimensional model of ELB which consists of demotivation and exhaustion.

Apart from the research into the structure and levels of ELB, scholars have also focused on exploring the connection between ELB and other psychological elements (Liu et al., 2025b, 2025c; Shao et al., 2019). For instance, Shen et al. (2015) pointed out that although buoyancy can help in buffering against burnout, over time, the experience of burnout can also damage buoyancy. The prolonged state of emotional exhaustion as well as the decrease in academic efficacy might erode the confidence of learners, reduce their perseverance, and affect their capability to regulate stress in an adaptive manner.

### 2.3. Academic Buoyancy

Academic buoyancy is a construct that has emerged from the broader field of positive psychology in education, emphasizing students’ everyday capacity to successfully deal with typical academic setbacks and challenges. Martin and Marsh (2008) defined academic buoyancy as the ability to overcome routine difficulties such as disappointing grades, test anxiety, homework overload, or temporary lapses in motivation. Unlike resilience, which generally refers to coping with major adversity or trauma, buoyancy addresses the more commonplace academic stressors that students encounter in their daily learning processes. In the field of SLA, academic buoyancy refers to providing learners with the capacity to negotiate the ups and downs of everyday language learning, sustain prolonged effort, and overcome setbacks on the path to L2 learning success (Yun et al., 2018).

Martin and Marsh (2008) pinpointed quite a few crucial elements that prop up buoyancy, and these are frequently encapsulated as the “5Cs”. These encompass confidence, which is another term for self-efficacy; coordination, referring to planning and organization; composure, meaning having low levels of anxiety; commitment, which is about persistence; and control, or adaptive attributions (Yun et al., 2018). Besides the concept of academic buoyancy, Martin and Marsh (2008) also scrutinized the proposed academic buoyancy hypothesis model by employing the Academic Buoyancy Scales (ABS). Yun et al. (2018) modified the ABS to tackle the hurdles that students faced in SLA, and this modification enabled the ABS to be more suitable for the context of SLA. The research also unambiguously illustrated that academic buoyancy was a strong predictor of students’ performance in their second language.

The existing empirical research has delved into the relationship among buoyancy, other psychological factors and academic achievement (Liu et al., 2024; Liu et al., 2025d; Li et al., 2023; Collie et al., 2017). In Putwain and Daly’s (2013) study, they revealed that students with high buoyancy levels were less likely to feel anxious during the test. It was also found that students with higher levels of academic buoyancy performed best when test anxiety was low or moderate. Martin et al. (2013) investigated the relationship between academic buoyancy and psychological risk factors (e.g., anxiety, failure avoidance, uncertain control). In addition, scholars have also reported the predictive effects of academic buoyancy on academic achievement (Aydın & Michou, 2020; Jahedizadeh et al., 2019). While buoyancy may not directly predict achievement, it appears to act as a moderator or indirect enabler by reducing vulnerability to burnout and fostering enjoyment. This nuanced role justifies its inclusion in configurational approaches.

### 2.4. Foreign Language Enjoyment

Foreign Language Enjoyment (FLE) is a positive emotion that has gained increasing attention in applied linguistics and educational psychology as part of the broader “positive psychology in SLA” movement (Dewaele & MacIntyre, 2014). Unlike transient pleasure or satisfaction, FLE refers to a more enduring sense of fulfillment and engagement that arises when learners feel stimulated, challenged, and rewarded in their language learning experiences. It encompasses cognitive, social, and emotional dimensions, making it a multifaceted construct that reflects both individual and contextual influences. FLE consistently emerges as central across both regression and fsQCA, underscoring the importance of intrinsic, rather than externally derived, enjoyment.

Scholars have stressed that FLE is not simply the lack of negative emotions like anxiety or burnout; instead, it is a distinct affective construct with its own unique antecedents and outcomes (Boudreau et al., 2018). Expanding upon the work done by Dewaele and MacIntyre (2014), later research has persistently pointed out two key dimensions: private enjoyment and social enjoyment. Private enjoyment alludes to the individual learner’s perception of progress, accomplishment, and cognitive stimulation while engaging with the foreign language. Social enjoyment, on the other hand, comes from interactions with teachers and peers, as well as from the overall classroom atmosphere that encourages collaboration and positive emotional exchange (Dewaele & Dewaele, 2017). Recent studies have indicated a third dimension, frequently called teacher-related enjoyment, which captures the unique part teachers play in molding learners’ affective experiences (Jin & Zhang, 2021). This dimension encompasses factors like teacher support, peer support, and intrinsic motivation in language learning. Collectively, these dimensions highlight the complex and relational structure of FLE, placing it as a construct influenced not just by learners’ internal states but also by their social and instructional settings.

In English learning contexts, high levels of FLE are often associated with greater willingness to communicate, stronger perseverance in the face of difficulties, and more effective long-term language acquisition (Dewaele & MacIntyre, 2016). By contrast, lower levels of FLE may leave learners more vulnerable to negative emotions such as burnout or anxiety (Liu et al., 2025a; De Smet et al., 2018). More recent studies also show that students who report higher levels of enjoyment are more likely to persist in language learning and achieve better outcomes (Khajavy & Aghaee, 2024; Zhao & Wang, 2023).

### 2.5. The Fuzzy Set Qualitative Comparative Analysis (fsQCA)

Fuzzy Set Qualitative Comparative Analysis (fsQCA) is a methodological approach that has gained traction in the social sciences for its ability to explore complex causal relationships. Developed by Ragin (2000), fsQCA combines the strengths of qualitative and quantitative research by employing set-theoretic logic to identify configurations of conditions that lead to specific outcomes. Unlike traditional statistical methods that typically estimate the net effects of individual variables, fsQCA recognizes that outcomes in social and educational contexts are often produced by combinations of factors that may vary across cases.

One of the crucial features of fsQCA is its employment of fuzzy sets. Fuzzy sets enable cases to possess varying extents of membership within a particular set, with the degree of membership ranging all the way from 0 to 1. This is in contrast to a mere binary classification that only distinguishes between presence and absence. Such a feature proves to be especially beneficial for educational research. In this field, constructs like buoyancy, burnout, and enjoyment are seldom absolute in nature. Instead, they tend to exist along a spectrum. By transforming raw data into fuzzy set scores through calibration, researchers are able to look into the intricate manners in which different levels of conditions engage in interactions to generate a specific outcome (Ragin, 2008).

In the realm of education, fsQCA has been progressively employed to look into the configurational impacts of psychological, motivational, as well as contextual variables upon student achievement (Chen & Chen, 2025). For instance, it is able to show how high degrees of enjoyment might make up for moderate degrees of burnout, or how resilience coupled with intrinsic motivation might foretell success even in challenging learning environments. These kinds of insights are frequently neglected by linear models, which presume symmetrical and additive effects.

Applied to English language learning, fsQCA is especially valuable because language achievement is shaped by multiple interrelated factors, including emotional, motivational, and contextual influences. The method enables researchers to capture the equifinality of learning pathways—that is, the idea that different combinations of conditions can lead to the same outcome, such as high achievement or sustained motivation (Fan & Wang, 2024; Xu & Feng, 2024). It also highlights causal asymmetry, where the factors leading to success may not simply be the inverse of those leading to failure.

**Figure 1:**
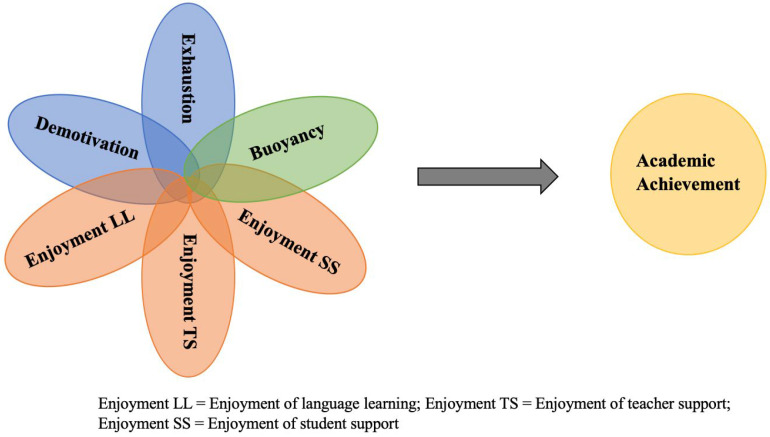
The configuration diagram of fsQCA.

By adopting fsQCA, the present study aims to move beyond traditional variable-centered analyses and to uncover the diverse configurations of burnout, buoyancy, and enjoyment that contribute to English learning outcomes (see Figure 1). This approach provides a richer, more holistic understanding of how affective and motivational factors interact in complex ways to shape learners’ academic trajectories. To this end, the study proposed three research questions:

RQ1: What are the relationships between students’ burnout, buoyancy and enjoyment in English learning?

RQ2: To what extent do burnout, buoyancy and enjoyment predict English academic achievement independently?

RQ3: Are there equifinal pathways of the configuration of students’ burnout, buoyancy and enjoyment in English learning that can result in high/low English academic achievement? If any, what are their configurational predictive effects?

## 3. Method

### 3.1. Participants

This study involved 640 senior high school students from a city in eastern China, including 273 (42.7%) male and 367 (57.3%) female participants. Among them, 201 (31.4%) were Grade one, 234 (36.6%) were Grade two and 205 (32.0%) were Grade three students. Every one of these students was a native Chinese speaker and was engaged in learning English as a compulsory subject as part of the national curriculum. To ensure that the sample was representative of the target population, a convenience sampling method was chosen. The questionnaires were distributed by the class head teachers, who also took the time to explain the purpose of the study to the students. The participants were given assurances that their involvement in the research was entirely voluntary and that their responses would be kept confidential, treated anonymously, and utilized solely for the purposes of academic research.

### 3.2. Instruments

The current research utilized a composite questionnaire that comprised two sections. The initial part encompassed demographic details like gender, age, and grade, along with the scores of the most recent English examination. Academic achievement was gauged through the scores of the standardized test conducted by the regional education department. To ensure the scores of different grades were comparable, z-score normalization was used for the standardization of test scores. The second part incorporated validated scales that separately evaluated students’ English learning burnout, academic buoyancy, and foreign language enjoyment. More specifically, it entailed three scales: the scale for measuring English learning burnout from Liu and Zhong (2022), the scale for measuring Academic buoyancy taken from Yun et al. (2018), and the scale for measuring foreign language enjoyment taken from Jin and Zhang (2021). These are all language-domain-specific measures, which can maintain consistency among the measures of burnout, buoyancy, and enjoyment in this study.

#### 3.2.1. English Learning Burnout Scale

Burnout was measured using the Senior High School English Learning Burnout Scale (SHSELBS) which was developed by Liu and Zhong (2022). This particular instrument comprises 10 items, and these items are further divided into two distinct dimensions. One of these dimensions is demotivation, which includes 6 items. An example of an item under this dimension would be something like “I feel emotionally drained by my English studies.” The other dimension is exhaustion, and it consists of 4 items. An instance of an item falling under this factor could be phrased as “I cannot effectively solve problems that arise in my English studies.” The responses to these items were rated on a scale that has five points, known as the Likert scale. When it came to prior validation, the scale exhibited robust psychometric properties. The values for these properties were as follows: α = 0.927, CFI = 0.943, TLI = 0.922, RMSEA = 0.078, and SRMR = 0.047.

#### 3.2.2. English Learning Buoyancy Scale

Academic buoyancy was assessed through Yun et al.’s (2018) four-item scale, designed for language learning contexts. Items were rated on a five-point Likert scale. A sample item is: Once I decide to do something for English learning, I persist until I reach the goal. The scale captures learners’ ability to recover from typical academic challenges. The construct of buoyancy also showed very good reliability and validity (α = 0.932, CFI = 0.999, TLI = 0.998, RMSEA = 0.033, SRMR = 0.006).

#### 3.2.3. English Learning Enjoyment Scale

Foreign language enjoyment was measured with a revised version of Jin and Zhang’s (2021) Foreign Language Classroom Enjoyment Scale. This scale has 17 items spread across three dimensions: enjoyment of teacher support (3 items, such as ‘The teacher is encouraging’), enjoyment of peer support (5 items, for example ‘We form a tight group’), and enjoyment of learning itself (9 items, like ‘In class, I feel proud of my accomplishments’). The instrument showed high validity and reliability (α = 0.883, CFI = 0.919, TLI = 0.901, RMSEA = 0.065, SRMR = 0.054).

### 3.3. Data Collection and Analysis

Data analysis was carried out across multiple stages. Initially, SPSS 29.0 was utilized for conducting descriptive statistics, reliability checks, normality testing, as well as correlation analysis. Following this, multiple regression analysis was employed to assess the independent predictive impacts of burnout, buoyancy, and enjoyment on academic achievement. Moreover, scale validity was further scrutinized via confirmatory factor analysis in Mplus 8.3. The model fit indices were evaluated according to the suggested thresholds by Hair et al. (2018), where RMSEA and SRMR were required to be below 0.08, whereas CFI and TLI had to exceed 0.90.

Eventually, fsQCA 4.1 was utilized to explore configurational effects. Prior to conducting the analysis, the raw data were calibrated into fuzzy sets. The calibration followed Ragin’s (2008) direct method. Empirical thresholds were determined using percentile anchors (95% = full membership, 50% = crossover, 5% = full non-membership) for each variable, ensuring that fuzzy scores reflected both theoretical expectations and empirical distributions. Specifically, the calibration procedure employs a logistic function to assign values to the specified anchor points, thereby transforming both the outcome and causal conditions into fuzzy membership scores. This conversion is conducted on the log-odds scale of full membership using the fsQCA 4.1 software. Table 1 shows the calibration criteria for each variable, thereby facilitating meaningful set-theoretic comparisons in the analyses that follow.

The dual use of regression and fsQCA is methodologically intentional. Regression identifies the net linear influence of individual predictors, suitable for examining main effects. In contrast, fsQCA uncovers configurational causality—how distinct combinations of conditions jointly yield outcomes. This complementarity allows us to explore both net effects and causal asymmetry, providing a richer understanding of emotional and motivational interactions. In educational psychology, where human behaviors are complicated and context-dependent, such a combination enhances validity and explanatory depth (Fiss, 2011; Schneider & Wagemann, 2012).

## 4. Results

### 4.1. Descriptive Statistics and Correlation Analysis of Burnout, Buoyancy and Enjoyment

Table 2 presents the descriptive statistics of the main research variables. The skewness and kurtosis showed that the data were normally distributed (Kline, 2016). The mean score for academic achievement was 92.7, with a standard deviation of 23, ranging from 0 to 150. Among the affective variables, exhaustion had a mean of 2.30 (*SD* = 0.90), while demotivation averaged 2.50 (*SD* = 0.84), both measured on a 1–5 scale. In contrast, buoyancy showed a relatively higher mean of 3.32 (*SD* = 0.91), suggesting moderate levels of learners’ capacity to cope with academic setbacks.

Regarding enjoyment, three dimensions were assessed: enjoyment of language learning (LL) had a mean of 3.39 (*SD* = 0.71), enjoyment of teacher support (TS) was highest at 4.40 (*SD* = 0.68), and enjoyment of student support (SS) had a mean of 3.78 (*SD* = 0.64). These results indicate that students experienced generally high levels of enjoyment, particularly from teacher support.

Table 3 illustrates the Pearson correlation coefficients among the variables. Academic achievement was significantly negatively correlated with exhaustion (r = −0.286, *p* < 0.01) and demotivation (r = −0.264, *p* < 0.01), indicating that higher levels of burnout and demotivation are associated with lower academic performance. In contrast, academic achievement was positively correlated with buoyancy (r = 0.201, *p* < 0.01), enjoyment of language learning (r = 0.331, *p* < 0.01), and to a lesser extent, with enjoyment of teacher support (r = 0.038, ns) and enjoyment of student support (r = 0.059, ns).

Notably, exhaustion and demotivation were highly positively correlated (r = 0.802, *p* < 0.01), suggesting they may co-occur as symptoms of language learning burnout. Both were significantly negatively correlated with buoyancy and all enjoyment dimensions. Conversely, buoyancy was strongly positively correlated with enjoyment of language learning (r = 0.700, *p* < 0.01), teacher support (r = 0.261, *p* < 0.01), and student support (r = 0.450, *p* < 0.01), highlighting its close connection with positive learning emotions.

### 4.2. Independent Predictive Effects of Burnout, Buoyancy and Enjoyment on Academic Achievement

The results of multiple regression analysis assessing the independent predictive effects of the variables on academic achievement are presented in Table 4. Among the predictors, enjoyment in language learning (LL) emerged as a significant positive predictor of academic achievement (β = 0.327, *t* = 5.379, *p* < 0.001). In contrast, exhaustion showed a significant negative effect (β = −0.128, *t* = −1.991, *p* = 0.047), suggesting that higher levels of exhaustion hinder academic performance.

Other variables, including demotivation, buoyancy, enjoyment of teacher support, and enjoyment of student support, did not significantly predict academic achievement, although the direction of effects for enjoyment of teacher support and student support was negative, which may warrant further investigation. The non-significant coefficients suggest that external enjoyment sources may not directly translate into measurable performance gains once intrinsic enjoyment is accounted for.

### 4.3. Configurational Predictive Effects of Burnout, Buoyancy and Enjoyment on Academic Achievement

#### 4.3.1. Necessity Analysis

After calibration, the necessity analysis was conducted to assess whether any individual condition is required for the outcome of high academic achievement. None of the conditions exceed the standard threshold of 0.90 for necessity (Schneider & Wagemann, 2012), indicating that no single condition is necessary for high academic achievement. However, academic buoyancy and enjoyment of teacher support demonstrate relatively high consistency (>0.70), implying they may play significant roles as part of sufficient configurations (see Table 5).

#### 4.3.2. Sufficiency Analysis

The fuzzy-set Qualitative Comparative Analysis (fsQCA) revealed several distinct combinations of conditions sufficient for the presence (HAA) and absence (~HAA) of high academic achievement (see Table 6). The overall solution for achieving HAA demonstrated acceptable reliability with a solution consistency of 0.805 and explained a substantial portion of the cases with a solution coverage of 0.610 (Ragin, 2008; Fiss, 2011). Five unique causal configurations (HAA1 to HAA5) were identified, each representing a different pathway to success.

One prominent pathway (HAA2), characterized by the presence of buoyancy and enjoyment of language learning, combined with the absence of exhaustion, demotivation, and enjoyment of teacher support, achieved a raw coverage of 0.505. This indicates this combination was present in over half of the cases exhibiting HAA (Rihoux & Ragin, 2009). It also showed a notable unique coverage (0.127), highlighting its distinct contribution. Similarly, HAA1, which additionally required the presence of enjoyment of student support and absence of enjoyment of teacher support, showed robust consistency (0.879) and substantial unique coverage (0.042). This pathway underscores the critical role of positive affective states like buoyancy and enjoyment in learning activities, coupled with the avoidance of negative states like exhaustion and demotivation (Martin & Marsh, 2008; Pekrun et al., 2002). HAA3, HAA4 and HAA5, featuring the absence of both Enjoyment SS and Enjoyment TS alongside the presence of buoyancy and Enjoyment LL, all demonstrated high consistencies (0.890, 0.861, 0.880) but lower coverages (0.011, 0.013, 0.003). Learners represented by these configurations appear to be autonomous and self-regulated, relying primarily on internal motivation and emotional stability rather than on social support.

Conversely, the analysis identified a single, highly consequential configuration (NHAA1) sufficient for the absence of high academic achievement (~HAA). This pathway consistently (Consistency = 0.809) explained a majority of the underachievement cases (Raw Coverage = 0.564; Unique Coverage = 0.564). It is defined by the co-occurrence of exhaustion and demotivation, alongside the absence of buoyancy and enjoyment of language learning. The irrelevance of Enjoyment TS and Enjoyment SS in this configuration suggests that regardless of teacher’s aide or peer support, the core combination of high exhaustion, high demotivation, low buoyancy, and low enjoyment in learning itself is a potent barrier to academic success (Salmela-Aro et al., 2009). The overall solution for ~HAA exhibited strong reliability (Solution Consistency = 0.809) and coverage (Solution Coverage = 0.564).

Overall, the fsQCA results underscore the complexity, asymmetry, and equifinality of affective and motivational influences on English learning achievement. Multiple distinct combinations of psychological and experiential conditions can lead to high achievement. The core elements across successful pathways consistently involve the presence of positive engagement (buoyancy, Enjoyment LL) and the absence of debilitating factors (exhaustion, demotivation). In stark contrast, underachievement is primarily driven by a singular, high-impact configuration dominated by negative states and the lack of core positive engagement, demonstrating asymmetry in the causal recipes for the outcome and its negation (Schneider & Wagemann, 2012; Woodside, 2013). These findings complement the regression results by demonstrating that linear effects alone cannot capture the interactive, non-linear patterns among burnout, buoyancy, and enjoyment. The configurational approach therefore provides richer explanatory power for understanding the psychological mechanisms underlying English academic performance among Chinese high-school learners.

## 5. Discussion

### 5.1. The Independent Predictive Effects of Burnout, Buoyancy and Enjoyment on English Academic Achievement

The regression analysis clarifies the unique contributions of the three affective constructs to students’ English academic achievement. Among all predictors, intrinsic enjoyment of language learning emerged as the most powerful and consistent facilitator of achievement. This finding corroborates both CVT and SDT: Chinese high school EFL learners who perceive high control and value (CVT) and experience autonomy and competence satisfaction (SDT) are more likely to sustain positive activating emotions such as enjoyment, which in turn drive deep engagement and achievement (Deci & Ryan, 2000; Pekrun & Perry, 2014). In practical terms, students who genuinely enjoy the process of learning English tend to invest more effort, persevere through difficulty, and apply self-regulated strategies that enhance performance.

Exhaustion, in contrast, had a marked negative impact on achievement, which implies that when energy resources are depleted, it directly impedes students’ capability to perform effectively. This aligns with studies on cognitive load and attentional resources (Schaufeli & Bakker, 2004) that indicate exhaustion restricts learners’ working memory capacity and diminishes their ability to focus, thereby reducing academic achievement. From a CVT standpoint, exhaustion reflects low perceived control and low value, which reduce motivation and lead to disengagement. Its negative effect highlights the importance of managing academic workload and promoting emotional recovery in exam-intensive environments.

Interestingly, demotivation and academic buoyancy did not significantly predict achievement when other variables were controlled. This non-significance does not negate their importance but suggests indirect or conditional roles. Demotivation may influence achievement primarily through its suppression of enjoyment and increase in exhaustion, whereas buoyancy likely functions as a moderator or mediator that strengthens the effects of positive emotions and buffers negative ones (Liu et al., 2025b; Li et al., 2023). In short, buoyancy helps maintain engagement but may not produce measurable performance gains unless paired with intrinsic enjoyment.

Finally, the non-significant or even slightly negative coefficients for teacher and peer enjoyment were unexpected but theoretically meaningful. Excessive dependence on external support may weaken learners’ sense of autonomy, which Deci and Ryan (2000) identifies as essential for intrinsic motivation. In some cases, strong teacher control or peer comparison might inadvertently reduce self-determined engagement. Thus, emotional well-being derived from external sources contributes to comfort and belonging but does not necessarily translate into higher performance unless internalized into self-regulated motivation.

### 5.2. The Configurational Predictive Effects of Burnout, Buoyancy and Enjoyment on English Academic Achievement

The fsQCA results expand upon the regression findings by revealing multiple equifinal configurations leading to high English achievement, alongside one dominant configuration predicting low achievement. This configurational evidence reveals the fact that the ingredients of success are not simply the contrary of failure. Across the five high-achievement configurations, intrinsic enjoyment of learning itself consistently appeared as a core condition, often combined with low burnout (low exhaustion and demotivation) and moderate to high buoyancy (see Table 7). These findings align with CVT’s assumption that positive activating emotions enhance learning when learners perceive both control and value, and with Deci and Ryan’s (2000) proposition that enjoyment derived from autonomy and competence is self-sustaining (Pekrun et al., 2002).

The most representative pathway (HAA2) combined high enjoyment of language learning and buoyancy with low burnout and little reliance on enjoyment of teacher support. This suggests that autonomous, resilient learners can achieve high outcomes even without strong emotional dependence on their teachers. In China’s exam-oriented context—where instruction tends to be authority-centered (Cheng, 2016)—such students may compensate for limited teacher-related enjoyment through self-regulated learning, goal setting, and persistence (Zimmerman, 2002).

Another prominent pathway highlighted the role of peer-related enjoyment. In this configuration, students combined high enjoyment in language learning with high enjoyment from peer interactions, alongside low exhaustion and demotivation, again without strong enjoyment from teacher support. This reflects the collectivist nature of the learning environment (Hofstede, 2001), in which peer scaffolding, cooperative learning, and shared academic goals provide a motivational boost (Gillies, 2016) that compensates for the absence of teacher-related enjoyment. The presence of peer enjoyment in this pathway suggests that, for some learners, the classroom peer network serves as an essential social resource (Wentzel, 2005) that enhances persistence and academic engagement, thereby contributing to higher achievement.

A smaller but highly consistent pathway (HAA3, HAA4 & HAA5) involved students who displayed both high buoyancy and high enjoyment in language learning but lacked enjoyment from both teachers and peers. This group appears to represent autonomous high achievers who are self-reliant in their approach to learning (Ryan & Deci, 2017), relying on personal resilience, intrinsic motivation, and disciplined study habits rather than on socio-emotional support from the learning environment. The reliability of this configuration indicates that, for certain learners, internal resources are sufficient to sustain high performance even in the absence of external motivational inputs (Duckworth et al., 2007).

Unlike those various high-achievement configurations, the analysis pinpointed just one prevailing pathway linked to the absence of high achievement. This particular configuration was typified by the concurrent existence of high exhaustion as well as high demotivation, together with low buoyancy and low enjoyment in the process of language learning. In these situations, external enjoyment sources, be it from teachers or peers, seemed to fail in reducing the negative effect of the combined emotional depletion, motivational decline, and scarcity of personal resources (Salmela-Aro & Upadyaya, 2014). Such a profile mirrors a condition where learners get entrapped in a cycle of disengagement and underperformance: exhaustion lowers the energy available for learning (Maslach et al., 2001), demotivation diminishes persistence, and low resilience obstructs recovery from academic setbacks. The striking difference between the variety of successful pathways and the singular, extremely harmful profile for failure highlights the asymmetric causal relations uncovered by fsQCA (Ragin, 2008).

When we consider the pathway-specific findings as a whole, it becomes clear that high English academic achievement has the potential to be maintained via various combinations of psychological and social factors. Each of these combinations represents a distinct equilibrium between internal resources, emotional states, and social supports. Certain students flourish owing to the joint existence of resilience and intrinsic enjoyment. For some others, it is the motivational advantages derived from peer interactions that play a crucial role. There are still others who benefit from autonomous learning strategies, which render external affective inputs less important. On the contrary, academic underperformance is closely associated with the simultaneous occurrence of multiple negative states along with the lack of essential personal resources. This implies that interventions ought to focus on dismantling this harmful configuration at the same time as they reinforce the positive combinations that have been pinpointed in successful pathways (Hiver et al., 2021).

## 6. Conclusions and Implication

This study enhances our comprehension of the way in which affective and motivational factors interact to impact English academic achievement within the context of senior high school students. Through the integration of regression analysis alongside fsQCA, the research has uncovered both the independent effects as well as the configurational effects pertaining to burnout, buoyancy, and enjoyment. The findings affirm that the intrinsic enjoyment derived from language learning stands out as the most potent individual predictor of achievement, whereas exhaustion tends to undermine performance. Furthermore, numerous different combinations of positive affective states together with personal resources—for instance, buoyancy, peer-related enjoyment, and intrinsic satisfaction—have been demonstrated to uphold high achievement, thereby exemplifying the principle of equifinality. Low achievement was mostly linked to a particular adverse situation marked by feelings of exhaustion, demotivation, as well as a lack of buoyancy and enjoyment. The difference in the predictors of success and failure highlights the significance of dealing with negative states, besides encouraging positive ones.

From a theoretical aspect, this research highlights the value of employing a configurational approach for comprehending academic achievement. Traditional regression models frequently presume symmetrical and linear connections, which pose a risk of concealing the multifaceted and interactive characteristics of psychological factors in the learning process. Through the application of fsQCA, this study illustrates that various combinations of burnout, buoyancy, and enjoyment can result in high achievement, thus reinforcing the principle of equifinality within educational psychology. This indicates that researchers ought to go beyond single-variable explanations and instead take into consideration the intricate, dynamic interplay of emotional and motivational states when developing theories about achievement emotions. Furthermore, the discovery that underachievement is mainly accounted for by a single harmful configuration emphasizes the principle of causal asymmetry, drawing attention to the fact that the paths to success and failure are not mere mirror images. These findings refine CVT by empirically confirming that emotional precursors to achievement operate asymmetrically—positive affective states (enjoyment, buoyancy) have a non-linear compensatory power, while negative states (burnout) exert dominant inhibitory effects. The integration with SDT further suggests that the satisfaction of autonomy and competence needs mediates these relationships. Hence, the study not only validates CVT in a language learning context but also contributes a refined, interaction-based model where affective configurations predict learning outcomes more accurately than single-variable effects.

From a pedagogical standpoint, the outcomes indicate that language educators as well as school administrators need to give priority to nurturing the intrinsic joy of learning activities, since it has been observed to predict achievement in a consistent manner across both independent and configurational analyses. Teachers can design autonomy-supportive activities (e.g., student-led projects), embed reflective self-regulation training, and introduce peer mentoring circles to simultaneously reduce exhaustion and enhance enjoyment. The classroom strategies that could be employed might involve crafting engaging and meaningful communicative tasks, promoting self-directed learning projects, and offering opportunities for students to experience mastery and competence. It is crucial to note that the enjoyment obtained solely from teacher or peer support was not enough to ensure higher performance. This suggests that social support ought to be utilized as a way to reinforce intrinsic engagement instead of being used as a replacement for it.

In summary, this study advances theory by showing how CVT can be extended through a configurational lens, capturing asymmetric and equifinal pathways to achievement. Methodologically, it demonstrates the added value of combining regression with fsQCA in educational psychology. Practically, the findings suggest that educators should prioritize nurturing students’ intrinsic enjoyment through engaging, meaningful learning activities, while simultaneously reducing exhaustion and demotivation. It is only when both sides of this emotional range are addressed that teachers and schools can foster sustainable accomplishment and psychological well-being in language learners. Specific strategies may include project-based learning, opportunities for autonomy, and stress-reduction interventions. Moreover, while teacher and peer support are important for emotional well-being, they should be leveraged to reinforce intrinsic engagement rather than substitute for it.

Despite its theoretical and methodological contributions, several limitations should be acknowledged. First, the study employed convenience sampling from a single urban region in eastern China, which may limit the generalizability of results to other cultural or educational contexts. Future research should replicate these findings using multi-site or stratified samples to enhance representativeness. Second, the cross-sectional design restricts causal inference. Longitudinal or experience-sampling methods could better capture the temporal dynamics of emotion and achievement. Third, while fsQCA provides configurational insights, it does not establish causal directionality. Future studies could integrate longitudinal QCA or hybrid modeling (e.g., SEM–fsQCA) to triangulate causal mechanisms.

## Figures and Tables

**Table 1:** Calibrations of the research variables.

Configurational Element	Fuzzy Set Calibrations		
	Fully In	Crossover	Fully Out
Academic Achievement [0–150]	129	93	55
Exhaustion [1–5]	4	2	1
Demotivation [1–5]	4	2.3	1
Buoyancy [1–5]	5	3.25	2
Enjoyment LL [1–5]	5	3.25	2
Enjoyment TS [1–5]	4.7	3.43	2.29
Enjoyment SS [1–5]	5	4.33	3

**Table 2:** Descriptive analysis.

	Mean	SD	Max	Min	Skewness	Kurtosis
Academic Achievement	92.70	23	150	0	−0.175	0.038
Exhaustion	2.30	0.90	5	1	0.658	0.108
Demotivation	2.50	0.84	5	1	0.275	−0.226
Buoyancy	3.32	0.91	5	1	−0.303	−0.052
Enjoyment LL	3.39	0.71	5	1.29	0.770	−0.028
Enjoyment TS	4.40	0.68	5	1	−1.290	2.798
Enjoyment SS	3.78	0.64	5	1.6	−0.292	0.362

**Table 3:** Correlation analysis.

	Academic Achievement	Exhaustion	Demotivation	Buoyancy	Enjoyment LL	Enjoyment TS	Enjoyment SS
Academic Achievement	-						
Exhaustion	−0.286 **	-					
Demotivation	−0.264 **	0.802 **	-				
Buoyancy	0.201 **	−0.476 **	−0.477 **	-			
Enjoyment LL	0.331 **	−0.626 **	−0.625 **	0.700 **	-		
Enjoyment TS	0.0380	−0.283 **	−0.307 **	0.261 **	0.343 **	-	
Enjoyment SS	0.0590	−0.308 **	−0.308	0.450 **	0.445 **	0.521 **	-

** Correlation is significant at the 0.01 level (2-tailed).

**Table 4:** Regression analysis.

	Unstandardized Coefficient	Standardized Coefficient	SE	*t*-Value	*p*-Value
Exhaustion	−3.261	−0.128	1.638	−1.991	0.047 *
Demotivation	−0.643	−0.024	1.759	−0.365	0.715
Buoyancy	−1.217	−0.048	1.351	−0.901	0.368
Enjoyment LL	10.666	0.327	1.983	5.379	***
Enjoyment TS	−2.198	−0.065	1.495	−1.471	0.142
Enjoyment SS	−2.805	−0.077	1.708	−1.642	0.101

* *p* < 0.1; *** *p* < 0.001.

**Table 5:** Analysis of necessary elements.

Configurational Element	Academic Achievement	
	Consistency	Coverage
Exhaustion	0.624	0.595
Demotivation	0.634	0.597
Buoyancy	0.712	0.705
Enjoyment LL	0.697	0.749
Enjoyment TS	0.734	0.601
Enjoyment SS	0.664	0.671

**Table 6:** Configurations for the presence and absence of high academic achievement.

Configurational Element	High Academic Achievement					~High Academic Achievement
	HAA1	HAA2	HAA3	HAA4	HAA5	NHAA1
Exhaustion	⊗	⊗		⊗	⊗	⬤
Demotivation	⊗	⊗	⊗	⮾		⬤
Buoyancy		⬤	⬤	⬤	⬤	⊗
Enjoyment LL	⬤	⬤	⬤	•	•	⊗
Enjoyment TS			⊗	⮾	⊗	
Enjoyment SS	⬤		⊗	⊗	⮾	
Conditions tested						
Consistency	0.879	0.819	0.890	0.861	0.880	0.809
Raw coverage	0.408	0.505	0.257	0.415	0.297	0.564
Unique coverage	0.042	0.127	0.011	0.013	0.003	0.564
Overall solution consistency	0.805					0.809
Overall solution coverage	0.610					0.564

⬤: presence of a core condition; •: presence of a peripheral condition; ⊗: absence of a core condition; ⮾: absence of a peripheral condition; blank: ‘don’t care’.

**Table 7:** Summary of major fsQCA configurations predicting high and low English achievement.

Pathway	Key Conditions	Outcome	Interpretation
HAA2	High Enjoyment_LL + High Buoyancy + Low Burnout	High Achievement	Purely intrinsic pathway
HAA1	High Enjoyment LL + High Enjoyment SS + Low Burnout	High Achievement	Intrinsic- extrinsic balanced pathway
HAA3, HAA4 & HAA5	High Enjoyment_LL + High Buoyancy + Low Enjoyment TS & SS	High Achievement	Intrinsic compensation pathway
NHAA1	High Exhaustion + High Demotivation + Low Buoyancy	Low Achievement	Negative affect-dominant pathway

## Data Availability

The data presented in this study are available on request from the corresponding author due to restrictions (privacy, legal or ethical reasons).
